# Direct Renin Inhibitor: Aliskiren in Chronic Kidney Disease

**DOI:** 10.5812/numonthly.3679

**Published:** 2012-12-15

**Authors:** Yoshiyuki Morishita, Eiji Kusano

**Affiliations:** 1 Division of Nephrology, Department of Medicine, Jichi Medical University, Tochigi, Japan

**Keywords:** Kidney Failure, Chronic, Renin-Angiotensin System, Aliskiren, Blood Pressure, Cardiovascular Diseases

## Abstract

The renin-angiotensin-aldosterone system (RAAS) plays pivotal roles in the pathogenesis of chronic kidney disease (CKD) progression and its increased complications such as hypertension (HT) and cardiovascular diseases (CVD). Previous studies suggested that aliskiren a direct renin inhibitor, blocks RAAS and may be effective for the management of CKD and its complications. This review focuses on the effects of aliskiren on CKD.

## 1. Introduction

Aliskiren is an orally active nonpeptide direct renin inhibitor, which acts by binding to the active site of renin (1). Aliskiren directly inhibits plasma renin activity (PRA), which acts at the initial and rate-limiting step in the renin-angiotensin-aldosterone system (RAAS), unlike the blockade of RAAS by angiotensin I-converting enzyme inhibitors (ACEIs) and angiotensin receptor blockers (ARBs) which cause a reactive rise in PRA (2).

RAAS plays pivotal roles in the pathogenesis of chronic kidney disease (CKD) progression (3). It has been reported that CKD is associated with increased risk of hypertension (HT) and cardiovascular diseases (CVD) (4-6). RAAS has been also shown to contribute to HT and CVD development in CKD (7, 8). Many clinical trials have demonstrated that blockade of RAAS by ACEIs or ARBs could prevent the development and progression of CKD and its complications such as HT and CVD (9-14). Recent evidences suggest that aliskiren would be effective for CKD management and its complications such as HT and CVD (15-21). This review focuses on clinical studies that have demonstrated the effects of aliskiren on CKD.

## 2. RAAS in CKD

RAAS plays pivotal roles in the pathogenesis of CKD (3). Among RAAS components, angiotensin II (ATII) mainly causes vasoconstriction in the efferent glomerular arteries rather than the afferent ones, which induces glomerular hypertrophy (22). ATII also causes vasoconstriction at glomerular capillaries, which affects the glomerular filtration rate (23). ATII also contributes to increase connective tissue production and extracellular matrix deposition (24). RAAS also plays pivotal roles in the pathogenesis of HT and the development of CVD in CKD (7, 8). The role of RASS in patients with hypertensive CKD was confirmed by the normalization of blood pressure (BP) following administration of an angiotensin antagonist, saralasin (25). Normally, volume overload and elevation of BP result in suppression of RAAS production. Since this feedback is often incomplete in CKD, patients with CKD often show HT and high or normal RAAS activity (7). Among RASS, ATII mainly contributes to the development of CVD. ATII has cellular effects that promote proliferation and hypertrophy of vascular smooth muscle cells and cardiac fibroblasts (26, 27). ATII also increases inflammatory mediators, which is an independent risk factor for CVD by directly increasing proinflammatory gene expression and activating oxidative stress, leading to progressive inflammation of the vascular endothelium (28-30).

## 3. The Effects of Aliskiren on CKD

Several clinical studies have demonstrated that aliskiren is effective on CKD (Table 1). Ito et al. reported that aliskiren (150-300 mg daily) decreased systolic BP (SBP) and diastolic BP (DBP) by 13.9/11.6 ± 11.6/9.7 (SBP/DBP, mean ± SEM) at 8 weeks in patients with CKD (serum creatinine: 1.3-3.0 mg/dL in males, 1.2-3.0 mg/dL in females) (19). Persson et al. reported that aliskiren (300 mg daily) treatment added to furosemide reduced the mean 24 h SBP by 6-8 mmHg on days 7, 14, and 28 compared to baseline value in 15 patients with diabetic nephropathy (eGFR: 75.5 mL/min/1.73m2) (16). UACR also decreased progressively compared to baseline value, with a 17% reduction on days 2-4, a 31% reduction on days 8-10, and a maximum reduction of 44% at the end of the treatment (day 28) (16). These results strongly suggested that aliskiren has beneficial effects for renoprotection and BP control in CKD.

**Table 1:** Clinical Studies on Aliskiren in Patients With CKD

References	Number of Patients	Study Duration	Intervention		Outcome					
					**Renoprotection**		**SBP/DBP (mmHg)**		**CVD**	
			**Treatment group**	**Control group**	**Treatment group**	**Control group**	**Treatment group**	**Control group**	**Treatment group**	**Control group**
Ito *et al.* (19)	40	3	aliskiren (150-300 mg daily)+ diuretics				-13.9/ -11.6			
Parving *et al.* (15)	599	6	aliskiren (150-300 mg daily)+losartan (100 mg daily)	placebo+losartan (100 mg daily)	-20% (UACRs)	0% (UACR)			-9.6 bursts/min (MSNA)	-0.7 burstsmin (MSNA)
Persson *et al.* (16)	15	1	aliskiren (150-300 mg daily)+ diuretics		-44% (UACRs)		from -6 to -8 (24 h MSBP)			
Siddiqi *et al.* (17)	25	1.5	aliskiren (300 mg daily)+existing drugs without ACEIs and ARBs	existing drugs			-27/ -13			
Moriyama *et al.* (18)	10	4	aliskiren (150 mg daily)+ olmesartan (10-40 mg daily)		-40% (UACRs)		no change			
Nakamura *et al.* (20)	36	6	aliskiren (150 mg daily)+ olmesartan (40 mg daily)	aliskiren (150 mg daily) or olmesartan (40 mg daily)	-541.3 mg/day (proteinuria) -14 mg/g Cr (L-ABP)	olmesartan: -304.0 mg/day (proteinuria) -7.5 mg/g Cr (L-ABP) aliskiren: -315.9 mg/day (proteinuria) -6.7 mg/g Cr (L-ABP)	-27/ -11.8	olmesartan:-19.6/ -8.3 aliskiren: -19.8/-8.7		
Morishita *et al.* (21)	30	2	Aliskiren (150 mg/day)+existing ACE inhibitor, ARB, CCB, α-blocker or centrally acting agents				-15/ -5		-62.5 pg/ml (BNP) -2.7 mg/l (hs-CRP) -38.7 U.CARR (d-ROM)	

Abbreviations: ARBs; angiotensin receptor blockers, ACEIs; angiotensin I-converting enzyme inhibitors, BNP; brain natriuretic peptide, DBP; diastolic blood pressure, d-ROM; diacron-reactive oxygen metabolite, hs-CRP; high-sensitivity C-reactive protein, L-ABP; L-fatty acid binding protein, MSBP; mean systolic blood pressure, MSNA; muscle sympathetic nerve activity, SBP; systolic blood pressure, UACR; urinary albumin-to-creatinine ratio

Parving et al. reported that treatment with aliskiren (150 mg daily for 3 months, followed by an increase in the dosage to 300 mg daily for another 3 months) added to losartan (100 mg daily) reduced the mean urinary albumin-to-creatinine ratio (UACR) by 20%; however, placebo did not reduce this ratio in 599 patients with hypertensive diabetic nephropathy (eGFR: 68.5 ± 25.7 mL/min/1.73m2 (aliskiren group), 66.8 ± 24.5 mL/min/1.73m2 (placebo group) (15). Furthermore, only small differences in BP (SBP: 2 mmHg lower (P = 0.07) and DBP: 1 mmHg lower (P = 0.08) in the aliskiren group) were seen between the aliskiren group and the placebo group by the end of the study period (15). Moriyama et al. reported that aliskiren reduced the UACR in 10 patients with CKD (eGFR 30-90 mL/min) (18). In that study, aliskiren (150 mg daily) reduced the UACR by about 40% after 16 weeks from baseline when it was added to olmesartan (10-40 mg daily); however, it did not change eGFR and BP throughout the study period (18). These results suggest that aliskiren may have renoprotective effects regardless of BP lowering effects.

Siddiqi et al. reported that aliskiren (300 mg daily) decreased SBP and DBP, as well as sympathetic activity, in 10 patients with CKD (eGFR 57 ± 22 ml/min/1.73m2) (17). SBP/DBP were reduced from 147/96 ± 10/7 to 120/83 ± 8/7 mmHg (P = 0.01) (17). The sympathetic activity quantified by assessment of muscle sympathetic nerve activity (MSNA) was reduced from 36 ± 8 to 26 ± 8 bursts/min (P = 0.01) (17). These results suggested that aliskiren could reduce sympathetic hyperactivity, which is often exhibited and contributed to the pathogenesis of HT and CVD in patients with CKD.

Nakamura et al. reported that the combination therapy of aliskiren (300 mg daily) and olmesartan (40 mg daily) caused greater reductions of SBP/DBP, proteinuria, and L-fatty acid binding protein (L-FABP), which is a marker of tubular injury, than monotherapy of olmesartan or aliskiren in nondiabetic patients with stage I or II of CKD over 6 months (20). In this study, the combination therapy of aliskiren and olmesartan reduced SBP/DBP from 157.3/89.3 ± 4.5/4.6 to 130.3/77.5 ± 2.3/2.7 mmHg, proteinuria from 1163.3 ± 239.5 mg/day to 622.0 ± 355.2.3 mg/day, and L-ABP from 32.2 ± 12.7 mg/g Cr to 18.2 ± 6.2 mg/g Cr. In contrast, olmesartan monotherapy reduced SBP/DBP from 155.8/89.5 ± 4.9/4.6 to 136.2/81.2 ± 5.0/3.5 mmHg, proteinuria from 1113.3 ± 201.7 mg/day to 809.3 ± 239.2 mg/day, and L-ABP from 33.1 ± 10.5 mg/g Cr to 25.6 ± 7.0 mg/g Cr, and aliskiren monotherapy reduced SBP/DBP from 157.6/90.2 ± 5.9/4.0 to 137.8/81.5 ± 4.0/2.3 mmHg, proteinuria from 1149.2 ± 264.9 mg/day to 833.3 ± 238.4 mg/day, and L-ABP from 32.2 ± 12.5 mg/g Cr to 25.5 ± 9.9 mg/g Cr (20). These results showed that the combination therapy of aliskiren and ARBs may be effective in patients with CKD.

Recently, we reported antihypertensive and potentially CVD-protective effects of aliskiren in patients with hypertensive CKD stage IV under hemodialysis (HD patients) (21). In this study, aliskiren (150 mg daily) significantly reduced SBP/DBP from 169.0/78.1 ± 20.1/12.0 to 153.7/73.0 ± 19.6/13.6 (P < 0.05) after two months (21). RAAS was suppressed with aliskiren regimen after two months (PRA: 3.6 ± 4.0 to 1.0 ± 1.5 ng/mL/hr, P = 0.004; angiotensin I (ATI): 1704.0 ± 2580.9 to 233.7 ± 181.0 pg/mL, P = 0.009; ATII: 70.2 ± 121.5 to 12.4 ± 11.5 pg/mL, P = 0.022) (21). Surrogate markers of CVD, such as brain natriuretic peptide (BNP), high-sensitivity C-reactive protein (hs-CRP), and an oxidative stress marker, diacron-reactive oxygen metabolite (d-ROM), were inhibited by aliskiren after two months (BNP: 362.5 ± 262.1 to 300.0 ± 232.0 pg/mL, P = 0.043; hs-CRP: 6.2 ± 8.1 to 3.5 ± 3.7 mg/L, P = 0.022; d-ROM: 367.0 ± 89.8 to 328.3 ± 70.9 U.CARR, P = 0.022) (21). These results suggested that aliskiren is effective for BP control and may have CVD-protective effects in patients with hypertensive HD.

We also investigated the long-term effects, safety, and tolerability in those patients with hypertensive HD (under submission). Among 25 patients, 11 patients continued with aliskiren treatment (aliskiren group). Ten patients were withdrawn from aliskiren treatment after 3 to 8 months due to symptomatic hypotension; some of those patients had their BP controlled with antihypertensives such as calcium antagonists (5 patients), α-blockers (1 patient), and β-blockers (1 patient) (aliskiren-withdrawn group). SBP/DBP decreased from 175 ± 18/80 ± 11 mmHg at baseline to 155 ± 19/76 ± 9 mmHg at month 20 in the aliskiren group. PRA, ATI, and ATII decreased from baseline to month 20 (PRA (ng/mL/h): 2.3 ± 2.6 to 0.3 ± 0.4 (P < 0.05), ATI (pg/mL) 909.1 ± 902.5 to 41.5 ± 14.8 (P < 0.05), ATII (pg/mL): 41.5 ± 45.8 to 11.0 ± 4.9 pg/mL (P < 0.05)). BNP and d-ROM showed tendencies to decrease from baseline to month 20 (BNP (pg/mL): 248.9 ± 197.2 to 203.7 ± 113.3, d-ROM AT (U.CARR): 386.6 ± 123.1 to 305.6 ± 67.4). On the other hand, in the aliskiren-withdrawn group, SBP/DBP decreased from 171 ± 13/80 ± 14 mmHg at baseline to 157 ± 26/77 ± 12 mmHg at month 20. Although PRA, ATI, and ATII decreased at month 2 with aliskiren treatment (Baseline-Month 2 PRA (ng/mL/h): 3.3 ± 2.9 - 0.9 ± 0.7 (P < 0.05), ATI (pg/mL) 999.1 ± 844.6 - 365.6 ± 211.6 (P < 0.05), ATII (pg/mL): 30.6 ± 36.7 - 8.4 ± 10.7 (P < 0.05), Ald (pg/mL): 97.2 ± 57.5 - 79.3 ± 35.7 (NS)), they increased above the baseline level by withdrawal of aliskiren at month 20 (PRA (ng/mL/h): 4.1 ± 3.3, ATI (pg/mL) 801.0 ± 1032.8, ATII (pg/mL) 26.1 ± 17.2). In addition, d-ROM showed a tendency to decrease from baseline to month 2 followed by a further decrease at month 20 (Baseline-Month 2-Month 20: d-ROM (U.CARR): 402.3 ± 69.0 (Baseline) – 362.3 ± 47.8 (month 2) – 336.3 ± 58.5 (month 20)); however, in one period, BNP (pg/mL) showed a tendency to decrease from baseline (425.3 ± 207.3 (Baseline) – 409.5 ± 287.8 (month 2)), but it did not decrease from month 2 to month 20 (412.9 ± 287.8). These results suggested that long-term treatment with aliskiren provides effective BP lowering and inhibition of CVD surrogate markers which are sustained over 20 months in patients with hypertensive HD.

## 4. Adverse Effects of Aliskiren on CKD

Hyperkalemia, which is a frequent concern in patients with HD regardless of medication use, is the primary danger from RAAS-blocking medications. The blockade of RAAS leads to a decrease in aldosterone levels. Since aldosterone has a central role in the excretion of potassium, RAAS blockers can cause potassium retention. Several clinical trials of aliskiren in patients with CKD tracked potassium levels (15, 16, 19, 21). No significant trend for increased hyperkalemia by aliskiren in patients with CKD was observed in these trials (15, 16, 19, 21). Although careful and periodical monitoring of plasma potassium level is required, these results suggested that the risk of hyperkalemia by aliskiren in patients with CKD is small. One study reported that 15% of patients with CKD had adverse events that were suspected of being related to aliskiren. The most frequently reported adverse events were mild to moderate nasopharyngitis, back pain, and dizziness (5% each) (19).

Several studies reported that mean trough plasma aliskiren concentrations increased with renal impairment (19, 31); however, an increase in exposure did not correlate with the severity of renal impairment (31). Moreover, renal clearance of aliskiren represents only a small fraction (0.1-1.0%) (2). Although these data suggest that initial adjustment of the aliskiren dosage is unlikely to be required in patients with CKD, careful observation of BP change is required for aliskiren treatment in patients with CKD because several studies have reported symptomatic hypotension with aliskiren in patients with CKD.

## 5. Conclusion

From previous studies, it is suggested that aliskiren has beneficial effects for renoprotection, the control of BP, and the prevention of CVD in patients with CKD. However, the choice of aliskiren as well as its use in the treatment of patients with CKD has to be carefully determined considering the possible adverse effects and potential interactions with other drugs being used together. Further high-quality studies that are well designed and have an adequate sample size are still needed to confirm the effects of aliskiren in patients with CKD.
